# The role of humanities in the medical curriculum: medical students’ perspectives

**DOI:** 10.1186/s12909-021-02555-5

**Published:** 2021-03-24

**Authors:** Loukia Petrou, Emma Mittelman, Oluwapelumi Osibona, Mona Panahi, Joanna M. Harvey, Yusuf A. A. Patrick, Kathleen E. Leedham-Green

**Affiliations:** Imperial College School of Medicine, Imperial College London, South Kensington Campus, Sir Alexander Fleming Building, London, SW7 2DD UK

**Keywords:** Humanities, Curriculum, Medical students, Medical education

## Abstract

**Background:**

The humanities have long been shown to play an important role in the medical school curriculum. However, few studies have looked into the opinions of medical students on the usefulness and necessity of the humanities as well as their extracurricular involvement with them. The aim of this study was to: a) understand medical students’ attitude towards the humanities in medical education and b) assess their understanding of the necessary qualities of doctors and how interaction with the humanities affects the development of such attributes.

**Methods:**

A mixed methods survey was designed to elicit demographics, engagement, interest and perspective on curricular positioning, and to explore how students ranked the qualities of a doctor. It was distributed to medical students of all year groups in the 6-year bachelor of medicine, bachelor of surgery (MBBS) course at Imperial College London.

**Results:**

One hundred nine fully completed questionnaires were received. No significant difference was found in engagement or interest in the humanities between genders. Students felt strongly that humanities subjects shouldn’t be assessed (71:18) though some felt it was necessary for engagement, while no consensus was reached on whether these subjects should be elective or not (38:31). The majority of students wanted more medical humanities to be incorporated into the traditional medical course with a preference of incorporation into the first 3 years. Junior medical students were more likely to rank empathy as a highly desirable attribute than senior students. Students provided qualitative insights into curricular positioning, assessment and value.

**Conclusions:**

This study provides the perspective of medical students on how and whether the humanities should be positioned in medical education. It may be helpful to medical schools that are committed to student involvement in curriculum design.

**Supplementary Information:**

The online version contains supplementary material available at 10.1186/s12909-021-02555-5.

## Background

The humanities, including the arts and social sciences, are important aspects of everyday medical practice, and they are usually incorporated early into medical education. The General Medical Council emphasizes the importance of understanding a patient’s psychological, social and cultural needs, alongside their pathology [1]. Extending medical education beyond the biomedical sciences and clinical skills is a core strategy in the development of professional values and behaviours, including professional identity formation [2]. Medical humanities may encourage a deeper understanding of patients’ illness journeys through promoting cultural inclusivity by use of longitudinal case studies for instance [3, 4]. The arts can nurture visual diagnostic and analytical skills [5], be an outlet for physicians at risk of burnout [6] and nurture the positive qualities of empathy, self-efficacy and efficient patient focused care [6, 7]. The humanities may also support the development of interpersonal skills required to take on leadership and management roles within the multidisciplinary team. Physician-led healthcare management has been shown to result in better performance financially and clinically [8, 9]. Humanities-based learning may also support the development of persuasive writing skills which support engagement in policy writing and global heath. Some essential tools needed to accomplish these are the humanity subjects of history, economics, law and sociology.

Previously researched stereotypes suggest a gender divide within the subjects, with men perceived as likelier to pursue sciences and women likelier to pursue the arts [10]. The UK’s Universities and Colleges Admissions Service (UCAS) service data reflects that science, technology, engineering and mathematics (STEM) subjects have been markedly less popular amongst female applicants, although women tend to predominate over most other fields [11], (Supplementary information, figure 1). However, it has been suggested that gender identity and society moderate these stereotypes and therefore students’ academic plans [12].

Many medical schools have introduced humanities-based courses into their curricula, which may be centering around poetry, prose, law and ethics. However, these elements are often elective in nature, predisposing to self-selecting students, which may bias follow-up surveys and questions to more positive outcomes [13]. These humanities are often introduced as isolated modules rather than integrated education, shying away from the holistic approach a modern doctor should embody and therefore prompting courses to be centered on the biomedical aspects of medicine [13]. Similarly, few studies have questioned how medical students would like the humanities integrated into their education, and their engagement with the humanities outside of these compulsory programmes, with little regard towards the temporal placement of these modules within the course [14].

Our study primarily aims to assess the opinions of medical students on the integration of the medical humanities into education courses. It focuses on factors such as the electivity, assessment and appeal of such courses and the practical implications of such additions to the curriculum such as the timing. This study also aims to analyse what students perceive as the main qualities and focal roles of a doctor, how factors such as gender influence this, and how this is affected by the students’ previous exposure and engagement with the humanities. Our study positions student involvement in curriculum design as important from a both values-based perspective [15] and from a more pragmatic perspective, recognising that student involvement is a precursor to intrinsic academic motivation [16].

This research was situated in a London-based university of science, technology and medicine, and as such students had limited exposure to the humanities, other than through elective components. The university has recently instigated a learning and teaching strategy [17] that has student engagement as a central focus, including a system of ‘student shapers’ to ensure that the curriculum is aligned to students’ needs and aspirations. The current study is part of the college’s commitment to incorporating the student voice into curriculum design.

## Methods

Our research is situated in a post-positivist paradigm, where we believe there are real measurable differences between groups of students, however complexity and context make it difficult to draw conclusions that are widely applicable. We also believe that knowledge is necessarily situated in its context, therefore readers will need to make locally relevant inferences by taking into consideration our description of context and comparing it to their own.

A mixed methods survey research study was conducted using a simultaneous nested design [18]. This survey employs an exploratory design, and as such any patterns or correlations emerging from the data are not deductively proving a priori hypotheses. Instead we are using an inductive process to see which patterns emerge, in order to generate hypotheses [19]. The questionnaire was composed of three parts; the first collected information regarding student demographics (self-identified gender, year of study, age, previous study or regular engagement with humanity subjects). The second focused on students’ viewpoints with regards to the humanities, specifically focusing on their relevance to medical practice and training. The third section focused on the integration of humanities into the medical course. Students could select multiple years that they thought the course should be included into and could give free-text answers for the reasons behind their choices. The questionnaire was created and distributed using©Qualtrics [20] (Supplementary information, Figure 2).

The questionnaire was trialed on a small selection of current medical students to ensure ease of completion, simplicity and relevance to the course, as no similar studies have been undertaken in the past. Ethical approval was provided by the Imperial College Medical Education Ethics Committee (MEEC1819–111).

All year groups from Imperial College School of Medicine who were students during the academic year 2018–2019 were eligible for the study with no exclusion criteria. The study ran from 1st October 2018 until the 2nd November 2018. Recruitment was via post-lecture announcements, direct approach and notification on the monthly newsletter. Participant information was included within the questionnaire and completion of the questionnaire qualified as consent for participation. The questionnaire required approximately 5–10 min to complete.

We analyzed the quantitative data for significant relationships using a combination of Kruskal Wallis and Mann-Whitney U tests with respect to age/year group/gender/previous humanities involvement using SPSS software version 26.0 (IBM Corp., Armonk, N.Y., USA). For the free-text responses, a modified version of consensual qualitative research (CQR-M) was used, involving one person coding, a second person auditing one in five answers and all authors co-constructing meaning and checking themes and categories against the underlying data until there was a good fit. Disagreements were resolved through mutual discussion. All responses were read to ensure no minority themes were missed. Coding continued until saturation, which was determined by no new themes arising [21].

## Results

**Fig. 1:**
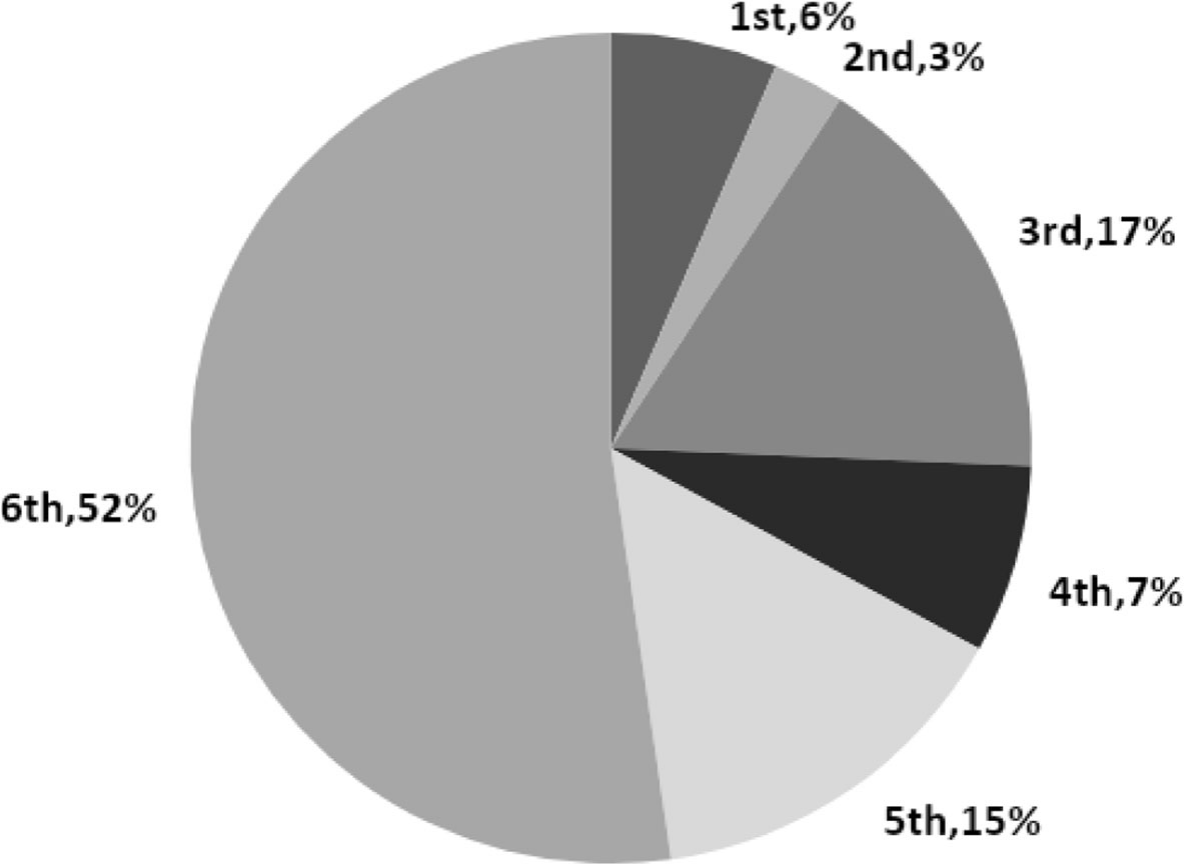
Pie chart of academic year distribution within the study population. Percentage of students per option stated

We received 123 responses of which 14 were excluded as incomplete, resulting in 109 fully completed questionnaires, (109/123, 88.6%). The average age of participants was 22, 51% identified as female, 46% as male, 1% non-binary and 2% preferred not to say. Participants' distributions across years are diplayed in Fig. 1. There was no significant difference between the population of this study and the population of the medical school in terms of gender, based on statistics provided by the admissions data.

In relation to the types of humanities different groups of students were interested in, those who engaged in the university’s elective, extra-curriculum, humanities programme were found to be more likely to have engaged with the visual arts (*p* = 0.031). There was a significant difference between senior (years 5 & 6) and younger medical students (years 1–4), with a smaller proportion of senior students engaging in performing arts compared to their younger counterparts (*p* = 0.005).

Medical students at Imperial College study an intercalated bachelor of science (BSc) degree in a selected module of their choice. One of the largest influences on interest in the humanities was found to be participation in the Medical Humanities, Philosophy and Law BSc. These students were significantly more interested in the humanities (*p* = 0.016) and likelier to support the integration of more humanities into the course (*p* = 0.044). Quantitative analysis of factors prior to medical school, including study of humanities subjects, did not show any significant impact on students’ views of the medical humanities.

### Medical students’ thoughts on which year of medical school humanities should be introduced into

**Fig. 2:**
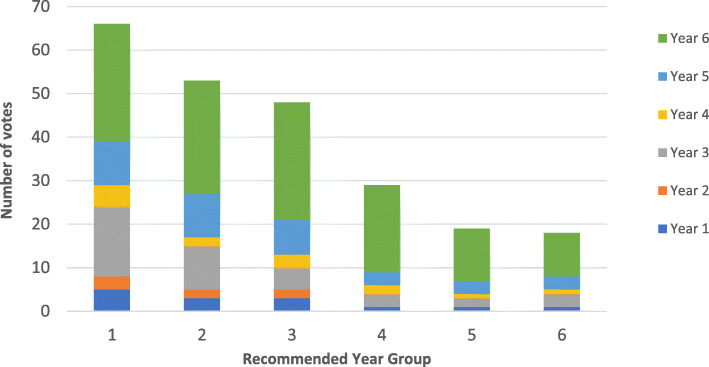
Recommended academic year for the introduction of a humanities course, split by current year group. (Relating to Q17: If more humanities subjects were introduced to the curriculum, in your opinion which academic year(s) would be most appropriate?) Bar chart split to represent response rates from different MBBS Year Groups

We found that students recommended the inclusion of additional humanities subjects in the first year most and final year least (Fig. 2). The majority of students (71.98%) recommended humanities incorporation in the earlier years (years 1–3).

Results from the qualitative questions, 17–19, are shown in Tables 1, 2 and 3, along with some exemplar quotations. Three main themes identified by students’ written responses included: (1) time, (2) use and (3) vertical learning. These themes were then separated into subthemes and these, along with their frequency and representative quotes are summarized in Table 1. Twenty-three responses were deemed unusable due to either being blank or incomprehensible.

**Table 1:** Question 17 “If more humanities subjects were introduced to the curriculum, in your opinion which academic year would be most appropriate?”

Theme	Subthemes	Quantity	Quotes
Time	More spare time in pre-clinical years More spare time in 3rd year More room for choice in 4th year Break from very science-focused younger years	22 14 10 5	“Early enough that there is still time to focus on it” “First proper clinical experience so allows for more holistic view of patient” “That’s the year you have a break from clinical medicine” “To get a taster view on why medicine is beyond the molecular science”
Use	Useful in clinical years to use in practice Pre-clinical years not enough empathy currently More open-minded in younger years	8 8 4	“Need clinical appreciation to best integrate” “Get an early start to open discussion and help keep the mindset open” “Earlier exposure would spark an interest sooner”
Vertical Learning	Useful to integrate early and late years Logistics Continuous learning	18 2 12	“Theory in early years, applied in clinical years” “Years in which lectures feature heavily and you’re mostly centrally based” “Core throughout entire degree”

**Table 2:** Question 18 “If humanities subjects were integrated into the course, should they be elective?”

Answer	Themes	Quantity	Quotes
Yes	Disinterest Unnecessary	17 11	“Not everyone has an interest or aptitude for humanities” “Forcing someone to do humanities benefits no one”
No	Produce well-rounded doctors Humanities are important Too many people would opt out People need to do it	3 8 11 4	“Helps to be a well-rounded person and enable to be a better doctor” “They are as important as other compulsory subjects” “Optional teaching is generally badly attended” “The people who don’t want to do it need it most”

**Table 3:** Question 19 “If humanities subjects were integrated into the course, should they be assessed?”

Answer	Themes	Quantity	Quotes
Yes	To ensure attendance and engagement Emphasise importance of humanities All course material should be examined	9 1 1	“Provide motivation” “They have the same importance and weighting as other parts of medicine” “If it is in the course it should be examined”
No	Too many exams already Humanities are subjective Engagement is more important than exams Humanities are not vital to role of doctor Exams detract from enjoyment Unfair to include in rankings	4 11 7 12 6 2	“There are enough exams to worry about” “Hard to examine, based on opinions and perspectives” “Allows people to engage with no pressure” “Important to consider but not an integral part of passing the course” “Assessment detracts from creativity and enjoyment” “Better to have a formative discussion or something where attendance is graded but not marked”

### Medical students’ opinions on whether humanities should be an elective module or not

We found that 31 students prefer humanities to be compulsory (‘No’) and 38 students showed preference in an elective course (‘Yes’). Forty students were indifferent and 64 written responses were unusable (Table 2). We found that the most common reasons for humanities to be an elective component were disinterest in the subject and that it was deemed unnecessary to the course. The themes, where given, are thematically summarized in Table 2.

### Medical students’ opinions on the assessment of humanities subjects

The final qualitative question related to whether the course, if introduced, should be assessed or not. In general, 18 students thought it should, 71 students thought it shouldn’t and 20 students were indifferent. Sixty-one written reasons were unusable. The reasons, where given, are thematically summarized in Table 3.

Regarding ranking the important qualities of a doctor, senior medical students (years 5 and 6) rated ‘importance of empathy’ lower (*p* = 0.0001) with the ‘ability to manage a patient’ ranked significantly higher (*p* = 0.001). Finally, senior students are significantly more likely to agree that humanities play an important role in the MBBS course (p = 0.001).

There was no significant difference between the genders in engagement or interest in humanities. The median values between all cohorts were similar and a study involving a larger sample size and perhaps qualitative reasoning behind answers given would be needed in order to draw meaningful conclusions.

## Discussion

There is some discourse regarding how and whether to integrate humanities into medical curricula at present [14, 22, 23]. Imperial College provides an epidemiology & sociology of medicine course in earlier years, an ethics course in 3rd year and a ‘dermatology and art’ teaching day in later years. There is also a Medical Humanities, Philosophy & Law intercalated BSc option in year 4.

The majority of students believe that additional humanities subjects would be better incorporated in earlier years. Qualitative comments suggested an integrated approach was preferred by some students, with some specifically requesting vertical learning throughout education. Participants highlighted the need to keep a broad-minded perspective of medicine in the pre-clinical years, when students may feel there is a lack of insight towards the broader aspects of a physician’s training outside scientific knowledge, a viewpoint shared by some critics of the standalone approach [14]. Furthermore, many older years state that there is greater time-flexibility during younger years, allowing for greater stimulation with humanities, which provides a break from the science-focus. In contrast, other participants view the humanities as a separate entity to medical education, which would take away from the scientific focus.

This disparity in students’ understanding of how the humanities relate to medical education was most starkly demonstrated in Table 2 where participants were split 55 to 45% in favor of humanities being a compulsory component of the course. The main reasons against compulsory integration were either the humanities being unnecessary to medical education or that students would be disinterested in the topics, which may be due to the lack of discussion around the utility of humanities in medicine [24]. Disinterest as a reason to avoid compulsory teaching could strengthen the idea that many students perceive the medical humanities as a separate entity from medicine. Conversely, those in favor of integration, cited its importance to a well-rounded education, and proposed that students in “need” of the skills obtained by the study of the humanities are those who would opt out from elective courses [23].

There were varying conceptions of what the humanities are, and how they relate to medical education, with some conceptualising the humanities as solely the arts and some with more complex conceptions that might include philosophy, sociology or history. This varied view of medical humanities role within the medical profession is shared in the literature with some emphasising its ability to help the physician at work, while others stress its role is outside the realm of academia [25, 26].

An area that many participants agreed upon is that engagement in humanities should be considered more important than assessment, citing the large number of exams already present in the medical school as a source of stress and concern [27]. Whilst outside the scope of this study, the role of exams in medical school was the largest detractor for assessing the humanities, yet those in favor of examination argue that students will only fully engage with a subject if it will be assessed. This evidently creates a challenge when trying to design a more holistic course which fosters learning through curiosity. Such antipathy towards examination is perhaps driven by concern over how the humanities could be assessed with the same objectivity analysed in more clinical and theoretical areas, with difficulties arising in the lack of measurable outcomes in areas such as empathy and professionalism, definition of terms and current pedagogical structures [28]. Expectancy theory would indicate that a lack of belief in the chance of personal success in such examination would lead to a decrease in motivation and effort invested towards these goals. Similarly, if self-perceived performance is not seen to correlate with assessment outcomes this disrupts the instrumentality of motivation, leading to further demotivation towards engagement with the topics [29].

Quantitative analysis found that there was no difference between genders in regards to interest in the humanities. This contradicts previous research and beliefs dictating that males tend to favour the more traditional STEM subjects, with females preferring humanities. Despite evidence to the contrary in this study, overall UCAS data still displays a major discrepancy between genders in regard to engagement in the humanities (Supplementary information, figure 1). This has largely been put down to societal pressures and a lack of role models in the field. However, this study shows that for those students who choose to study medicine, a science that combines humanistic care with science-based clinical practice, there is no significant difference in engagement or interest in the humanities. Whilst difficult to interpret, this may indicate that when exposed to similar societal pressures, which pushed these individuals to pursue a medical vocation, there is no inherent difference between genders.

The comparison between senior and junior students indicated a disparity wherein senior students were less likely to engage with the humanities. This is indicative of the culture of medical school where students approaching final exams often struggle to continue extracurricular pursuits they developed in their younger years. As described previously by Shapiro et al. [30], humanities-based learning is not valued by all students which prompted us to investigate further how these students value humanistic professional qualities. A focus on academia and in particular the management of a patient is especially important to senior medical students as the focus of their written and practical examinations. These may have influenced the results of senior students rating management significantly higher in contrast to their junior counterparts, who overall deemed empathy more important. This also resonates with the work of Hojat M et al., which suggests that the empathy of students decreases in later clinical years [31]. This may be an indication of a change in priority as students move towards a more goal-oriented outlook on medicine, focusing on final exams and imminently approaching careers, as opposed to the holistic view that can be afforded in younger years. Older medical students are thus framed as approaching their education in more of a survivalist manner, focussing on future career experiences. A lack of value attributed to non-clinical skills could parallel more negative responses towards incorporation of the humanities into medical education in more senior years as valence is seen to be a key factor in motivation stimulation.

Unsurprisingly, there is correlation between doing the humanities and thinking positively about the humanities, however it is difficult to see whether engagement in the humanities drives satisfaction or vice versa. There are elective opportunities for students more inclined towards the humanities within the medical curricula, for example the extra-curricular courses and the Humanities BSc. However, this doesn’t lean towards a more integrated nature afforded by the humanities, or elective modules more directly related to medicine. It also demarcates that the humanities are only related to medicine in a purely accessory capacity, something which was highlighted as problematic by the students surveyed in this study. As valence is intrinsically linked to motivation, this attitude detracts from students’ willingness to engage with the humanities. This is perhaps linked to wider biases in medical education, as seen through use of learning tools such as Bloom’s taxonomy [32]. A study of evidence-based medicine learning objectives across medical schools in Canada and the US showed that overall learning objectives more commonly focussed on knowledge, comprehension and application, rather than the higher levels of Bloom’s taxonomy, such as analysis, synthesis and evaluation [33]. It is possible that study of the humanities would allow access to the higher levels of Bloom’s taxonomy, but that these are pervasively not valued in medical education. This could constitute a form of social conditioning as students’ opinions become aligned with that of seniors and peers.

This study has provided the views of 109 medical students on the humanities in medical education, allowing us to explore a range of perspectives. The study is limited by being situated in a single institution and our sample is unlikely to be representative. As such our conclusions may not be generalisable, rather transferrable with an appreciation for the characteristics of participants and context. Our analyses of variance, for example between genders, rely on there being sufficient numbers in each group rather than representativeness, and therefore these findings may be more robust.

## Conclusion

Overall this study found there is a significantly large cohort of the student body that desire medical humanities to be more integrated into a traditional medical course, with many seeing it as an important concept in younger years. These feelings towards the humanities extended throughout the genders self-identified in the research cohort and independent of previous humanities exposure, contrary to prior literature. However, the utilisation of this topic in an examinable format still remains an element of contention for students feeding into the larger area of assessment being necessary for engagement. Despite this, it is evident that many students see the medical humanities as an important part of the medical curriculum which is currently being underserved. This study provides a good starting point for discussion and further research on the role of medical humanities in medical studies and how it may subsequently influence the qualities of a doctor.

## Supplementary Information

**Additional file 1: Supplementary Figure 1**. Percentage of undergraduate applicants that are female, over the past 10 years. Created using data from: UCAS Analysis and Insights 2018 [11]. **Supplementary Figure 2**. Questionnaire distributed to students using Qualtrics software

## Data Availability

The datasets used and analysed during the current study are available from the corresponding author on reasonable request.

## Abbreviations

MBBS: Medical bachelor and bachelor of surgery
UCAS: The universities and colleges admissions service
STEM: Science, Technology, Engineering and Medicine
CQR-M: Consensual qualitative research for simple qualitative data (modified)
BSc: Bachelor of science
