# Supplementary material for: The role of humanities in the medical curriculum: medical students’ perspectives
Source: BMC Med Educ. 2021 Mar 24;21:179. doi: 10.1186/s12909-021-02555-5 (PMC7992827; doi:10.1186/s12909-021-02555-5)

***Supplementary figure 1:*** *Percentage of undergraduate applicants that are female, over the past 10 years. Created using data from: UCAS Analysis and Insights 2018 (11).*


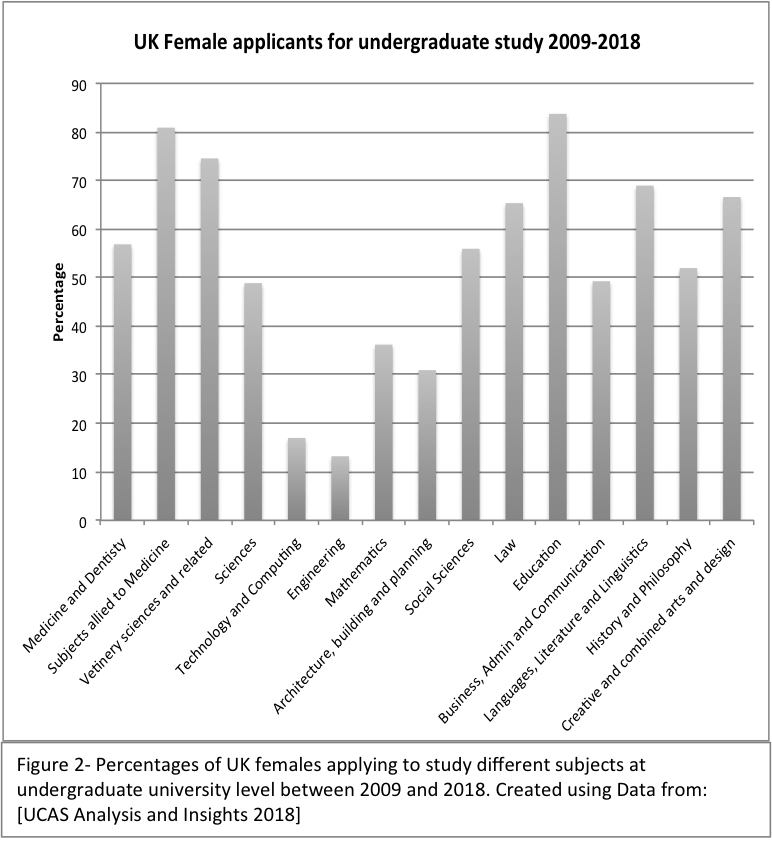


UK female applicants for undergraduate study 2009-2018


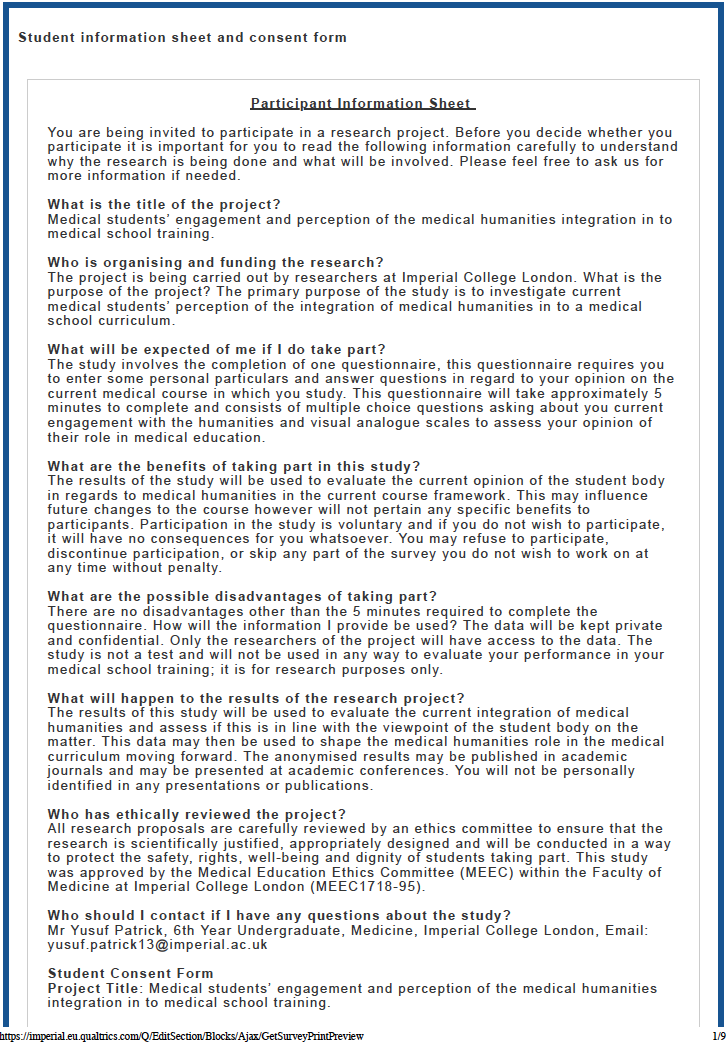

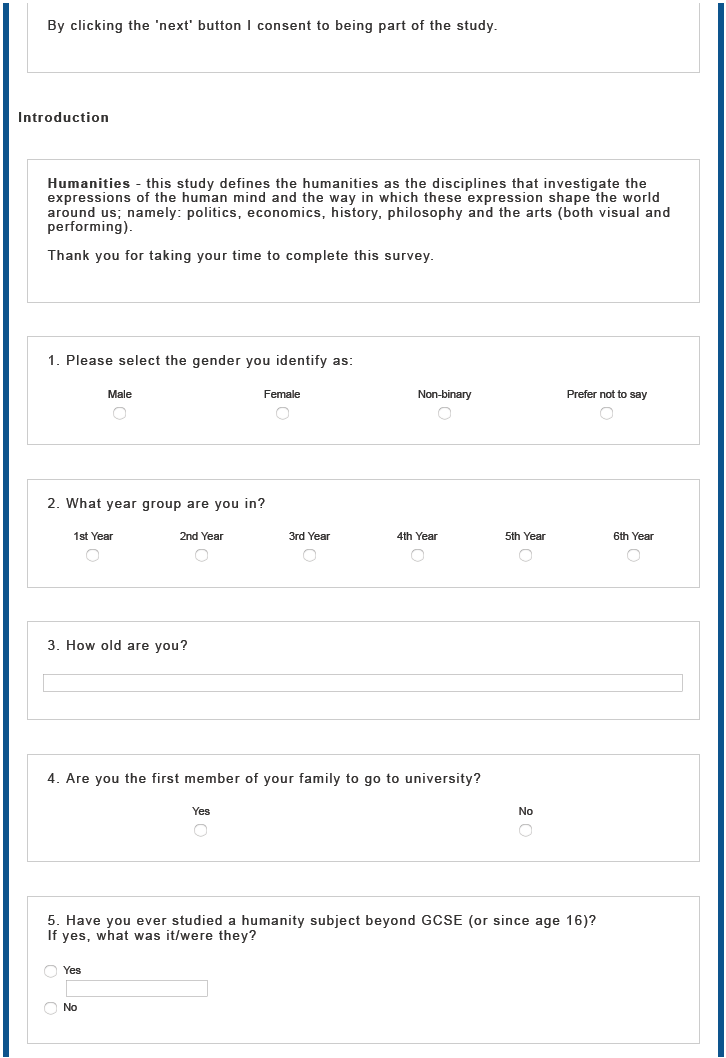

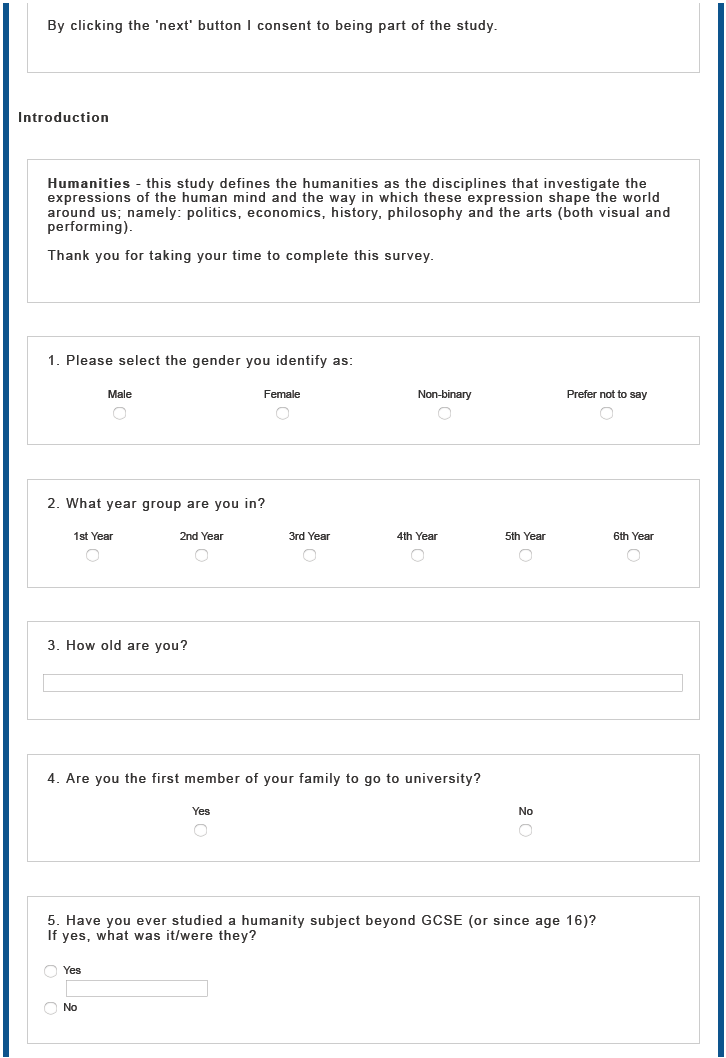


***Supplementary figure 2****: Questionnaire distributed to students using Qualtrics software.*


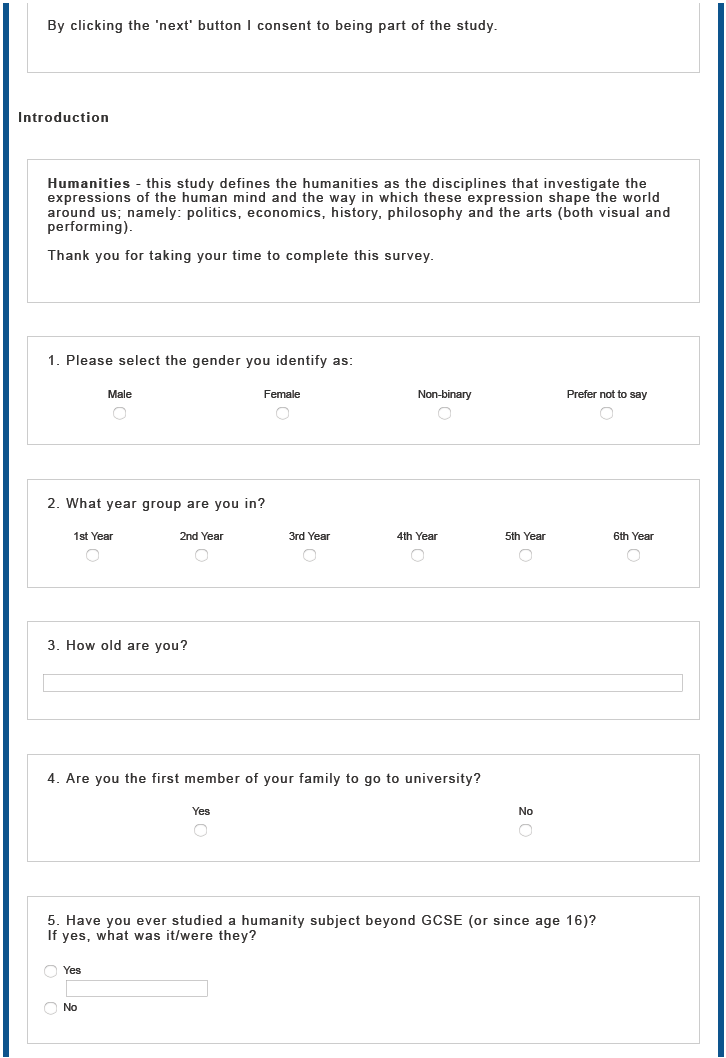

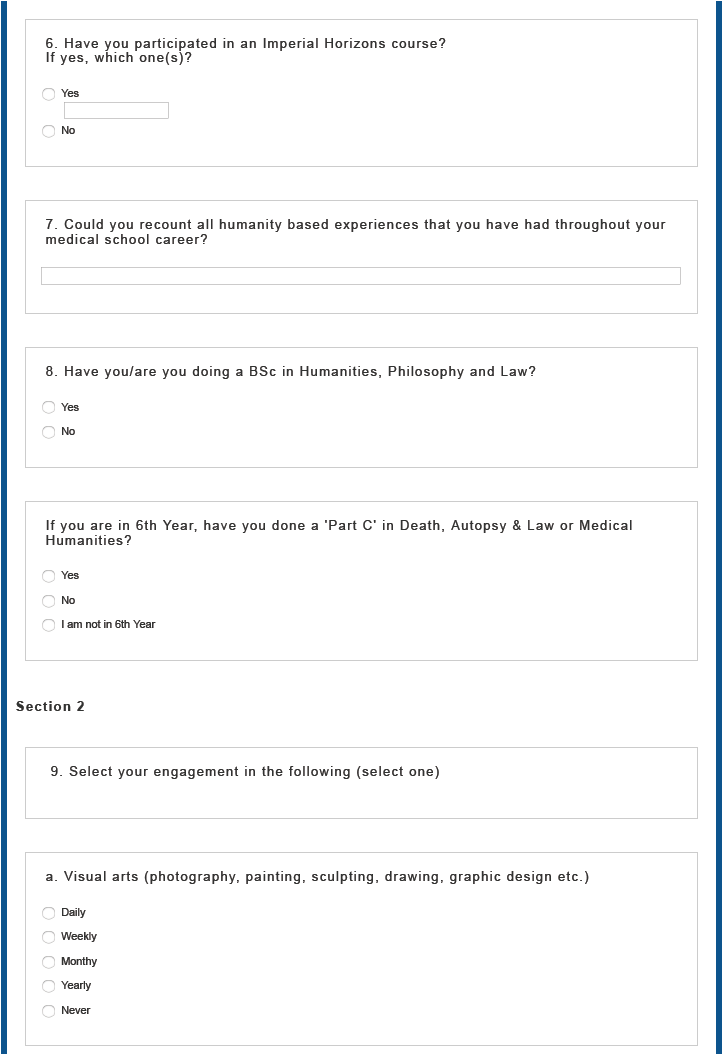

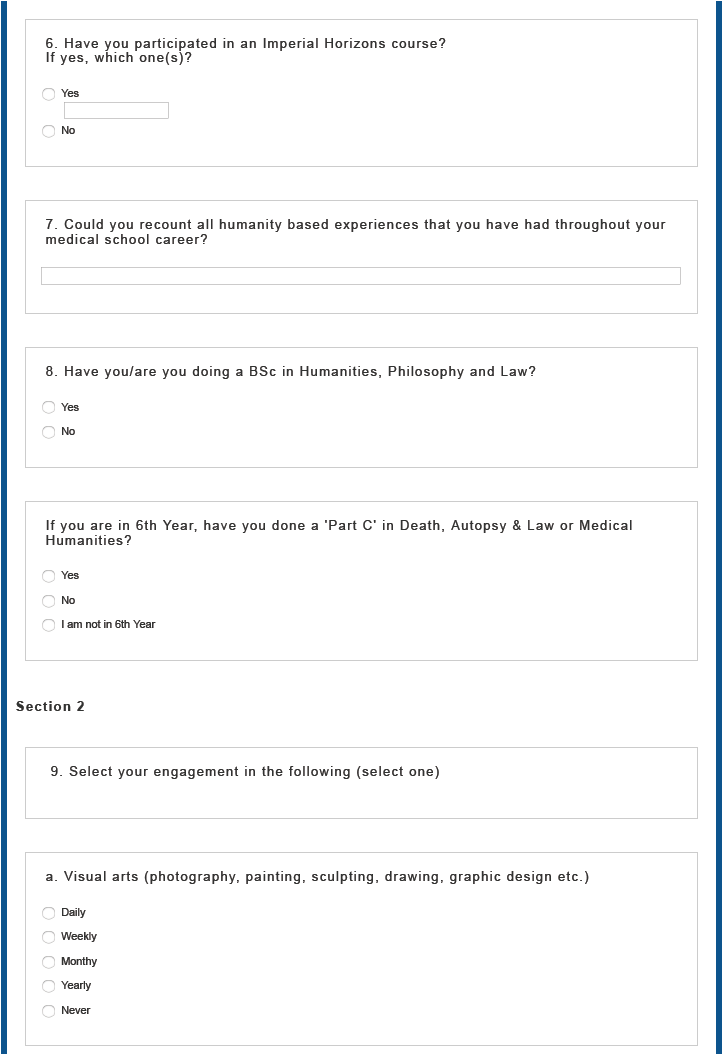

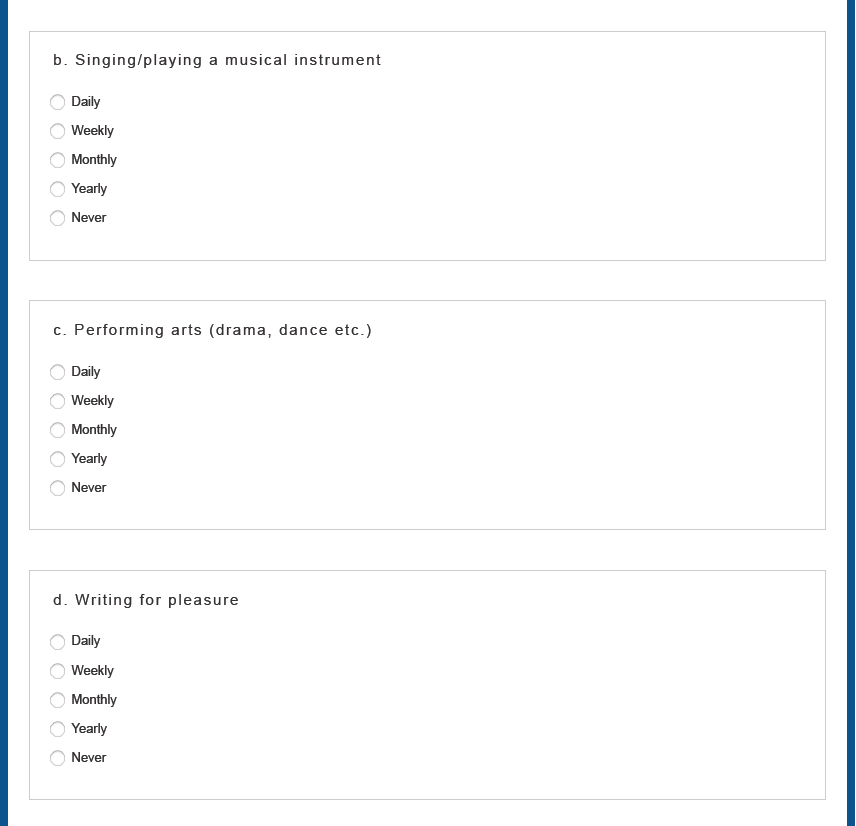

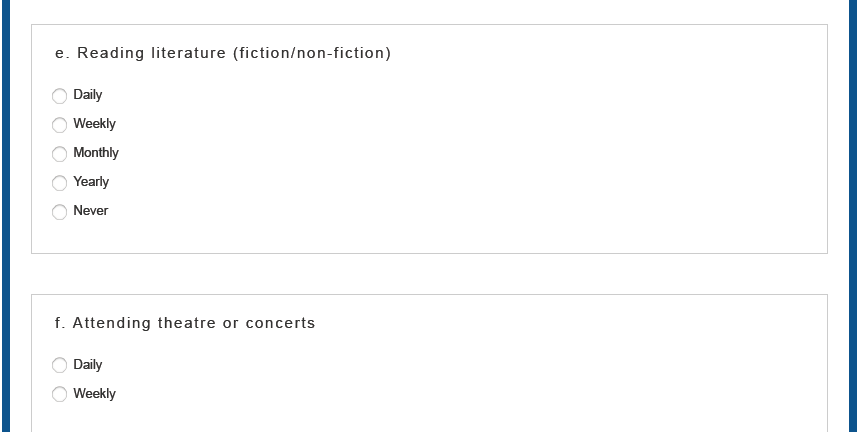

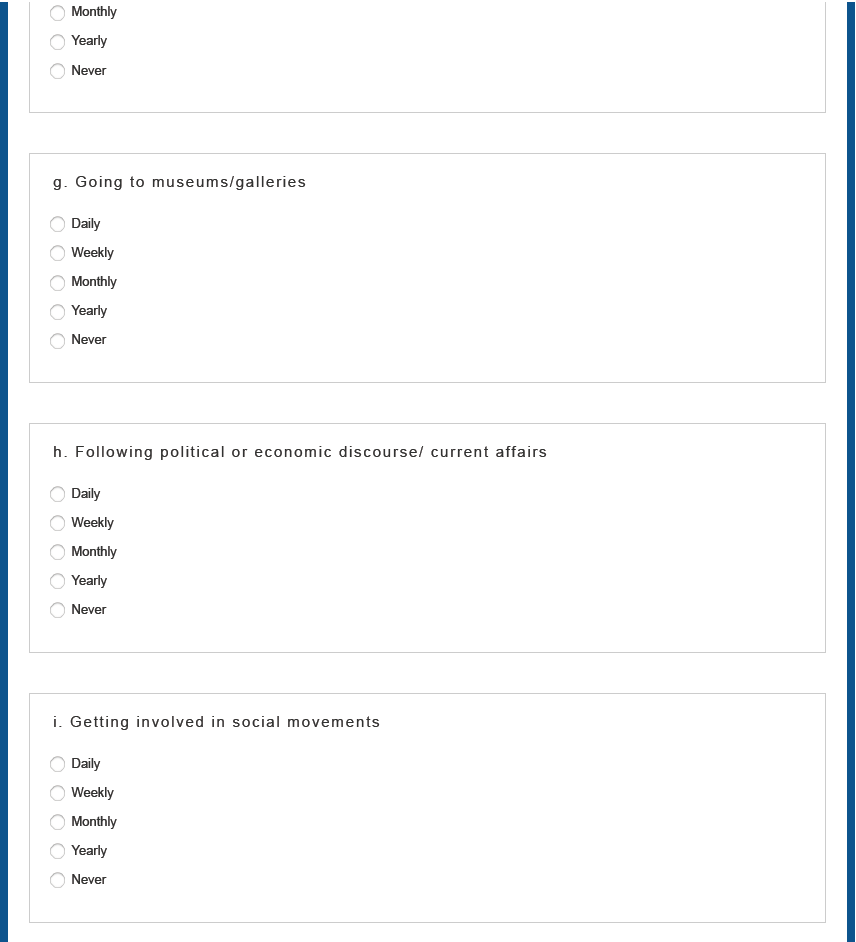

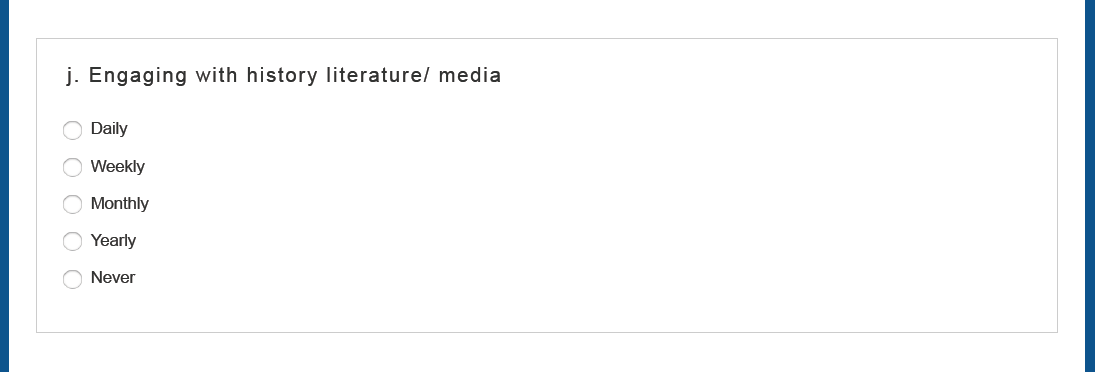

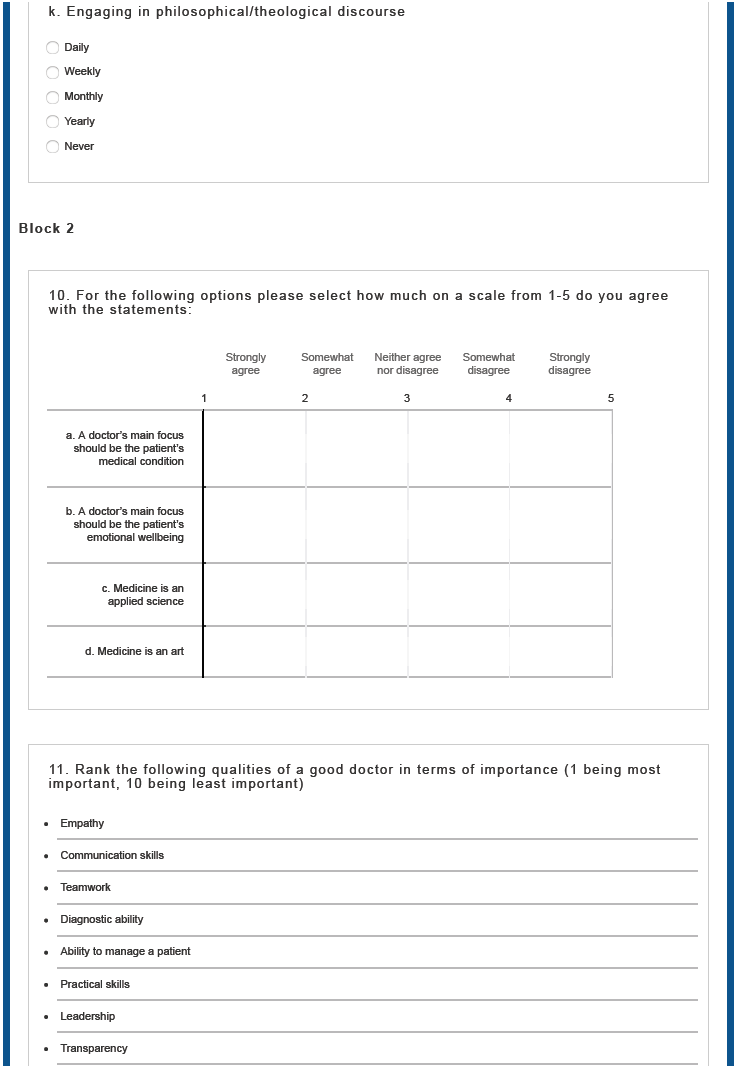

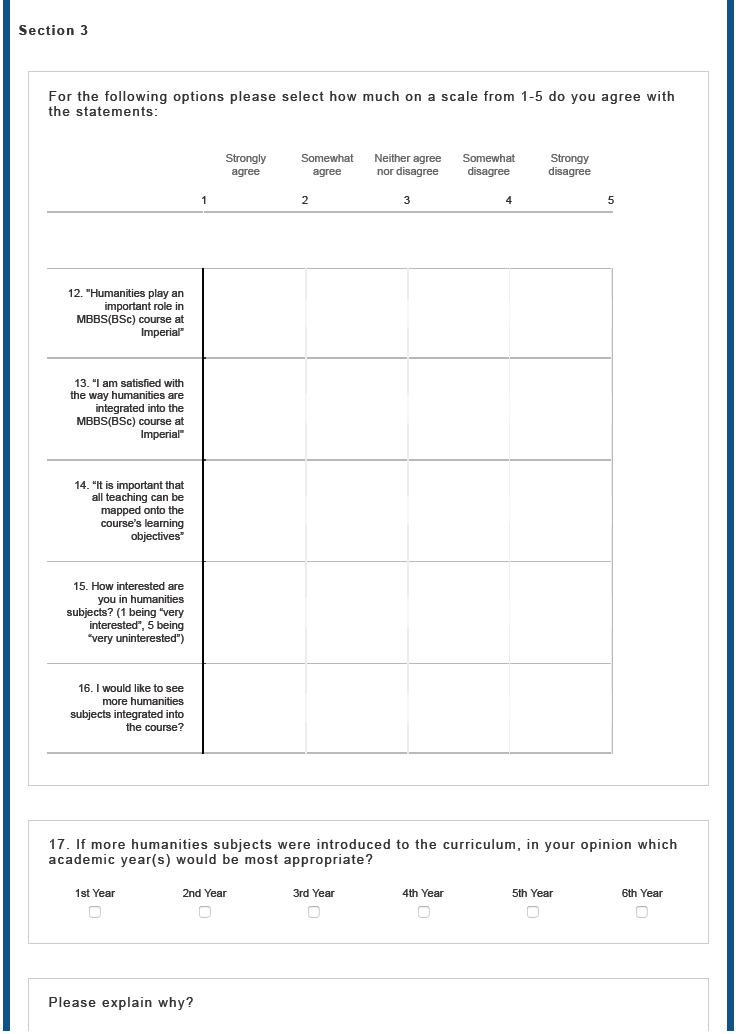

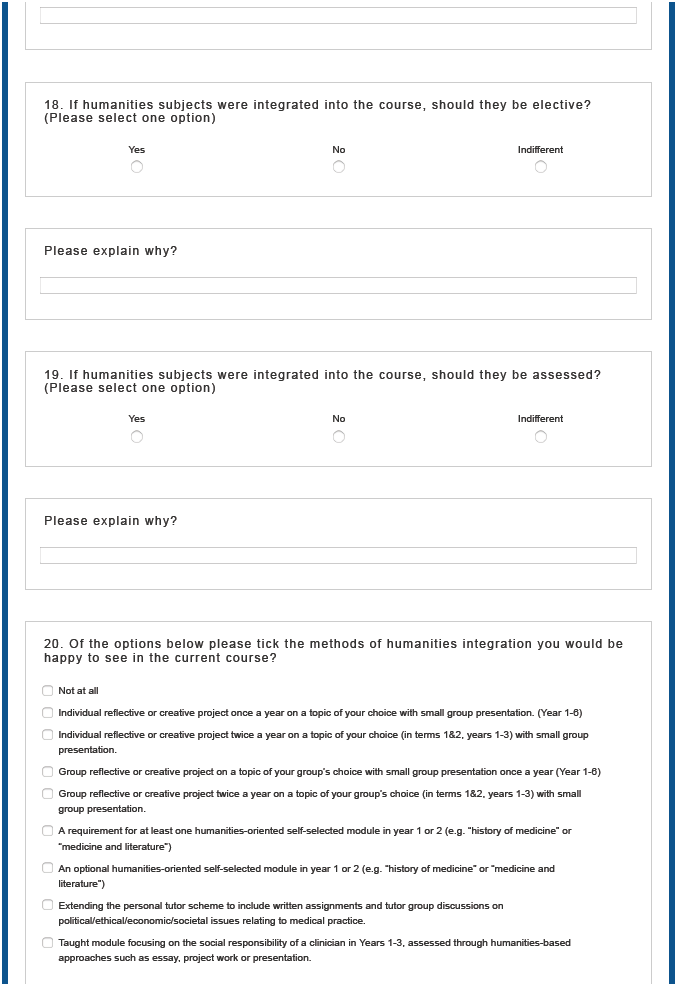

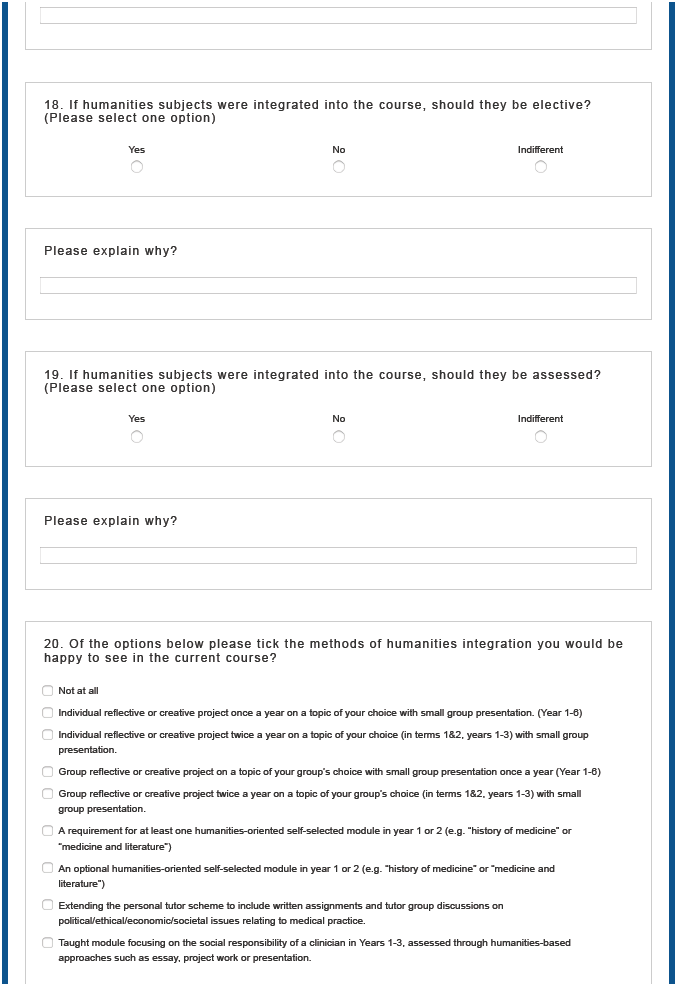

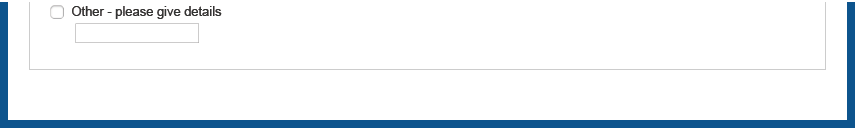

Supplement: Supplementary file 1 — Additional file 1: Supplementary Figure 1. Percentage of undergraduate applicants that are female, over the past 10 years. Created using data from: UCAS Analysis and Insights 2018 [11]. Supplementary Figure 2. Questionnaire distributed to students using Qualtrics software [file 12909_2021_2555_MOESM1_ESM.docx]
